# Estimating Population Parameters using the Structured Serial Coalescent with Bayesian MCMC Inference when some Demes are Hidden

**Published:** 2007-02-12

**Authors:** Greg Ewing, Allen Rodrigo

**Affiliations:** 1 Allan Wilson Centre for Molecular Ecology and Evolution; 2 Bioinformatics Institute, University of Auckland, Private Bag 92019, Auckland, New Zealand

**Keywords:** Coalescent, Migration, Markov Chain Monte Carlo, Bayesian Inference, Serial Samples, Measurably Evolving Populations

## Abstract

Using the structured serial coalescent with Bayesian MCMC and serial samples, we estimate population size when some demes are not sampled or are hidden, ie ghost demes. It is found that even with the presence of a ghost deme, accurate inference was possible if the parameters are estimated with the true model. However with an incorrect model, estimates were biased and can be positively misleading. We extend these results to the case where there are sequences from the ghost at the last time sample. This case can arise in HIV patients, when some tissue samples and viral sequences only become available after death. When some sequences from the ghost deme are available at the last sampling time, estimation bias is reduced and accurate estimation of parameters associated with the ghost deme is possible despite sampling bias. Migration rates for this case are also shown to be good estimates when migration values are low.

## Introduction

Frequently when we estimate the parameters associated with an island population model with migration using the structured Kingman coalescent (Kingman, 1982a,Kingman, 1982b), it is assumed that we sample all the demes in the population. In practice, sampling every deme may not be possible; frequently the data is just not available. It may be the case that we do not even know how many demes there are for the population of interest. Previous work on hidden (or ghost) demes was done by Beerli (2004). In that study he considered 3 subpopulations, 2 with sampled data and one that was not sampled. The study included a range of migration rates to and from the ghost deme. Estimates were then obtained with simulated data using a Maximum Likelihood approach with the program MIGRATE (Beerli and Felsenstein, 2001, 1999), both with and without the ghost deme. Beerli’s results are summarised in Table 1. It was found that estimated population sizes of observed demes tend to be biased when the estimation model is different from the true model.

In this paper we extend the work in Beerli (2004) to Bayesian estimation of coalescent-based population parameters using sequence samples obtained serially over time. In particular, we look at the ability of the Bayesian method presented in Ewing et al. (2004) to resolve population sizes when there are hidden or ghost demes with serial samples. We further extend this to the case where there is a single sample from the ghost deme at the last time point. This case can arise in HIV patients, for example when some tissue samples and viral sequences only become available after death (Wong et al. 1997; Nickle et al. 2003). The baseline model we use is a 2-deme model with samples coming from only one deme at 3 different times and possibly from the second deme at the last time point. In Beerli (2004) migration rates were also estimated. However, Beerli’s study used maximum likelihood and was for isochronous (i.e., non-serial sample) data only.

By focusing on population size, we can restrict our models to just 2 demes in order to reduce the times needed for inference and simplify analysis. We look at the problem of estimation with and without the ghost deme and consider the related problem of inference under the assumption of a ghost deme when the true model has none.

The rest of this paper is set out as follows. In the next section we discuss the details of the simulation datasets and the procedures used to estimate parameters over these datasets. In Section 3 we outline the main results and show appropriate summary statistics. Comparison to the general findings of Beerli (2004) are mentioned and we present results under the ghost deme model while the truth contains no such deme. We also consider how often the truth would be rejected from a 95% Highest Posterior Density interval (HPD). Finally we discuss these results with regard to practical approaches to applying this type of inference to real problems.

## Methods

We now discuss the specific parameters used for simulation and estimation. Data were simulated under the coalescent with the appropriate model, then parameters inferred with the same code that was presented in Ewing et al. (2004); Ewing and Rodrigo (2006). Table 1 shows the different data generation and estimation models considered. We produce a genealogy from the coalescent prior based on the given true parameters and then generate simulated sequences. The length of the simulated sequences was relatively short (250 base pairs) to allow for faster likelihood evaluations and to better replicate the uncertainty of branch lengths and topology that we see in real data of greater length. The simulated data consists of 3 time points equally spaced with 10 sequences per time point per deme. The distance between time points was 7.5 × 10^−2^ expected number of substitutions per unit time with a normalised mutation rate of 1. This produced small trees (30 sequences) but the population size can still be accurately inferred from such a dataset as shown in Ewing et al. (2004) and Drummond et al. (2002). Following the notation in Ewing and Rodrigo (2006), population size was parameterised as *θ_i_* = *N_i_t_g_* where *N_i_* is the effective population size in deme *i* and *t_g_* is the generation time. We set *t_g_* = 1 and use *θ*_1_ = *θ*_2_ = 0.1 for both deme 1 and 2 respectively, throughout this study. Migration rates *λ* to and from ghost deme were symmetric with units of expected number of migration events per lineage per expected substitution with values 0, 1, 2, 5, 10, 20 and 50. This covers the interesting ranges of high migration (*λ*≫1/*θ*), low migration (*λ*≪1/*θ*) and intermediate migration (*λ*≈1/*θ*). A *λ*= 0 indicates that the ghost deme does not contribute to the coalescent or genetic diversity.

**Figure 1:**
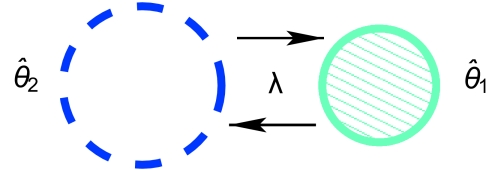
Schematic representation of the model. The dashed circle represents the hidden and unsampled ghost deme.

For each set of simulated data parameters, 51 datasets were generated and the parameters inferred with the Bayesian MCMC (Ewing et al. 2004; Ewing and Rodrigo, 2006). Estimation was done with constrained symmetric migration rates. That is *λ̂*_12_ = *λ̂*_21_ (Figure 1). However, the same assumption on *θ̂*_1_ and *θ̂*_2_ was not made, so in general *θ̂*_1_ ≠ *θ̂*_2_ Mutaion and genealogy were also estimated, but we omit the results due to very good estimation that is independent from the population parameters, (see Drummond et al. (2002), Ewing et al. (2004)). The MCMC chains were run for 10 million generations which we sampled every 1000 generations. For estimating without the ghost deme, the run lengths were much shorter: only 1 million generations with sampling every 200 was enough for good effective sample sizes (> 500). We check convergence by looking at topology, mutation rate and tree height. If these parameters encompass the truth then we consider that the chain is in the stationary distribution. Further checks for convergence included estimation of effective sample size (ESS) from the integrated autocorrelation time *τ*(IACT) (Geyer, 1992) and visual checks. In cases where the chain did not appear to converge (i.e., ESS less than 100), the chain length was increased until the ESS was at least 100 or more. Only a few chains and parameter ranges yielded ESS lower than 500.

Finally we generated 51 sets of zero-migration coalescent genealogies (i.e., models where no ghost deme is present) as in Beerli (2004). For this model *θ*_1_ = 0.1; We estimated the true parameters assuming the absence of a ghost deme, and under the assumption that the ghost deme is present.

**Figure 2:**
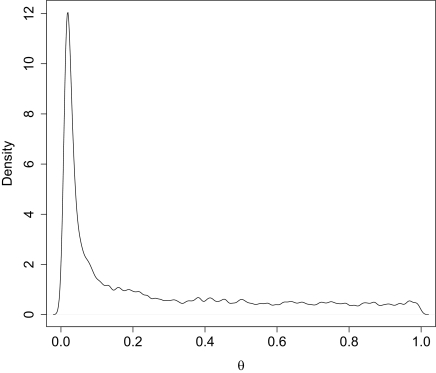
Marginal posterior density for *θ̂*_1_. The data was generated with *λ* = 0 and then the parameters estimated with a 2 deme model. There is a long tail that can often dominate the HPD intervals despite the well defined mode.

Mode estimators from the posterior are used as estimators due to the long tails that are common for marginal posterior density plots (Figure 2) and provide both lower bias and lower sensitivity to priors. The mode is estimated by noting the posterior density is inversely proportional to the distance between a fixed number of ordered values. We use 4% of the samples for the ordered values and take a median for the estimate. We have found that this provides very good estimates and is accurate when compared with Gaussian kernel posterior density estimates (Ewing et al. 2004). We only include estimates for sampled demes due to the high level of uncertainty for un-sampled demes, i.e., we do not report *θ̂*_2_ for Cases I and II.

95% Highest Posterior Density intervals were estimated in a similar fashion. We simply look for the shortest distance between ordered samples that encloses 95% of the samples. This method fails if the posterior is multi-modal, and in all cases marginal posterior densities were checked visually to ensure that they are unimodal.

In this study, we counted the proportion of 95% HPD’s that enclosed the true value of *θ*_1_, as well as the the value of *θ*_1_ + *θ*_2_, where appropriate. The latter value of *θ*_1_ + *θ*_2_ represents the true effective size under an equivalent panmictic model (Nagylaki, 1980). Hence under high migration rates, it is expected the estimated *θ*_1_ for the sampled population will approach *θ*_1_ + *θ*_2_

## Results

The major results of this study are summarised in Tables 1 and 2. Effective Sample Sizes were about 500 for worst-case parameters with the majority of runs significantly higher than this. We see from the Table that there is a trend of poor inference when we estimate under the wrong model. We overestimate *θ̂*_1_ without the hidden deme when *λ* > 0 and we underestimate *θ̂*_1_ when the hidden deme is included incorrectly. These general trends are in line with those observed by Beerli (2004).

### Case I(a): Estimation Without A Ghost Deme When *λ* = 0

First we consider the base case when there is no ghost deme and estimation is performed without a ghost deme. Due to the smaller number of parameters we expect this to be the case of best performance. In this case the true value of *θ̂*_1_ is enclosed in the HPD interval 100% of the time. The mean of the modes shows no evidence of bias. Visual inspection of individual runs also shows that performance is excellent, with fast convergence to equilibrium and efficient mixing with ESS > 1000. Since this case is essentially simple estimation under a correct coalescent model, these results are not surprising and echo those of others (Felsenstein, 1992; Drummond et al. 2002).

### Case I(b): Estimation With A Ghost Deme With *λ* = 0

**Figure 3:**
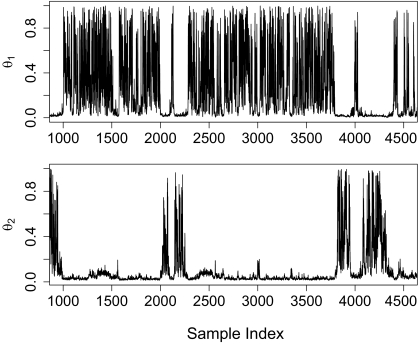
Traces of *θ*_1_ and *θ*_2_. The clear jumps correspond directly to genealogy migration patterns. Most coalescent events are in one or the other deme, corresponding directly with either poorly determined population size (~ 0 coalescent events) or a well defined population size.

**Figure 4:**
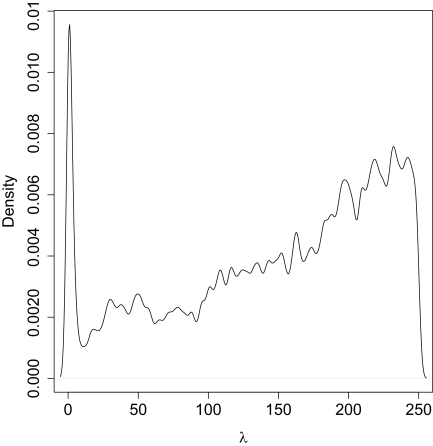
Marginal Posterior Density for migration. There is a clear peak at zero. The rest of the posterior is noisy due to the poor mixing over this large range and the ESS for this parameter was the lowest for this run at 150. The prior is a flat bounded prior from 0 to 250.

For this section we consider the performance when the data is generated with *λ* = 0 or without a ghost deme, but we assume that a ghost deme exists for estimation. The estimation of *θ̂*_1_ assuming the existence of a hidden deme showed substantial downward bias of the mode estimator with a mean of 0.02. In Beerli (2004), the general trend of a downward bias is also observed. This sets the general theme for the results: when we estimate under an incorrect model we get poor inference performance. 95% HPD intervals are very wide and are not significantly different from the prior (Figure 2). A short section of a trace for both populations is shown in Figure 3 and we see a “mode” shift in the chain. At some point the parameter *θ̂*_1_ is well defined and low compared to the bound while *θ̂*_2_ jumps between the bounds. In other parts of the plot we see that the opposite is true and *θ̂*_2_ is well defined while *θ̂*_1_ is moving between the bounds. If we inspect the genealogies for both cases we see that at any given time a majority of the coalescent events occur in one or the other deme. Thus there is insufficient information to estimate the population size of just one deme at a given time. The transitions between these modes is quick and efficient, and ESS is large for this parameter. Some of this bias may be due to the fact that the migration marginal posterior density has substantial weight on migration rates > 0 (Figure 4). If the chain spent more time in the close-to-zero migration rate, it would not bias the estimate of *θ̂*_1_ since in this case the posterior density reduces to the plain coalescent.

In Figure 4 the peak close to *λ* = 0 indicates that there is substantial probability around this true value. But this peak is small in terms of the total probability that it represents and the marginal density indicated that this is not distinguishable from high migration. We now need to consider if the lack of support for low migration, when the truth is zero, is due to mixing. The chain can only slowly remove migration events and in turn lower migration rates, thereby only mixing down to the lower migration values rarely. Even the recolour move described in the appendix in  Ewing and Rodrigo (2006), does not appear to help the mixing. A better move (MCMC kernel proposal) would be to change the migration rate and regenerate the migration nodes as a single move. We have not implemented this move for this study. We did inspect the 51 marginal posterior densities and we noted a small number that rejected low migration. When we extend these runs^1^ (× 2–5 longer), it was found that in most cases the peak at *λ* = 0 would manifest itself, and in a majority of cases this peak was the mode. This is a clear indication that the migration in particular does not mix properly despite the high ESS. This can be understood from the small chance of getting close to small values of *λ* with a random walk over permissable values and indicates that there could be some sensitivity to the prior bound. Some exploratory chains were run with larger priors with the result of a reduction in the peak at zero and reduced mixing.

Furthermore we attempted to explore the use of a flat prior in log space or a Jeffreys prior on all parameters with the effect of hugely increasing the mixing of the *θ̂*_1_ and *θ̂*_2_ parameters by casing longer waiting times between the flips. Migration mixing also suffered very badly and informative results were not produced. In general much of the above trends appear to exist, as the chain still spends significant time away from the zero migration rate. It should also be noted that MCMC runs with poor mixing did not change the *θ̂*_1_ and *θ̂*_2_marginal posterior densities as compared to chains with improved mixing.

This scenario illustrates the potential bias a flat bounded prior has on Bayesian inference. As we increase the upper bound on *λ* we increase prior support for a non-zero migration rate. With the current prior, a non zero migration rate is never rejected from a 95% confidence interval. We will discuss how we might overcome these limitations in section 4. Prior sensitivity analysis was not carried out except as mentioned above and formal hypothesis testing would need more thorough treatment.

### Case II(a): Estimation Without A Ghost Deme With *λ* > 0

When the migration rate is not zero and we estimate without a ghost deme the acceptance of the true value for *θ̂*_1_ immediately drops and the mode estimator becomes biased. This trend continues with increasing migration rate and the mode estimator for *θ̂*_1_ tends to *θ*_1_ + *θ*_2_. Also we reject the true value of *θ̂*_1_ from a HPD interval in more than 50% of the runs while we accept *θ̂*_1_ = *θ*_1_ + *θ*_2_ in more than 80% of the runs. So in effect we infer the population size of the single deme to be the sum of both demes. This is indeed the behaviour of the structured coalescent under high migration (Notohara, 1993; Hudson, 1990). It is surprising that the bias is strong even for very low migration rates and indicates that model misspecification of the type tested here gives rise to large errors in inference.

### Case II(b): Estimation With A Ghost Deme With *λ* > 0

If we include the unobserved deme in the estimation process, this bias is strongly suppressed. We see from Table 2 that when we estimate with a ghost deme mode estimators of *θ*_1_ are not far off from the truth. We accept the true value as expected and we reject the false values at about the same rate as the base case or case I(a) (the first column of Table 2 of case I). We have omitted the summary statistics for parameters of the ghost deme itself due to the fact that the marginal posterior densities for *θ*_1_ varie only slightly from the prior. That is, it is flat and extends out to the bounds. Marginal migration posterior densities are also generally uninformative. With low migration rates (5 or less) there was support for a region smaller than the prior bounds, but it was still diffuse. At high migration rates the marginal posterior density did exclude low migration rates, but was otherwise flat out to the bounds.

### Case III: Estimation With A Few Sequences From The Ghost Deme

**Figure 5:**
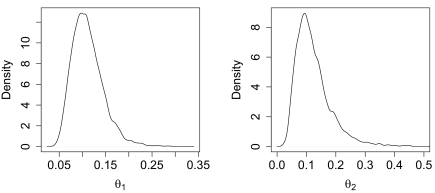
Marginal posterior density for *θ*_1_ and *θ*_2_ when there are a few sequences collected from the ghost deme. Both posterior densities are well resolved.

When we have just a few sequences (10) from the ghost deme at the most recent sample time only, the power of the method increases significantly. From Table 2 we see that even this small amount of data has reduced the bias seen under estimation with a ghost deme. With this data we also have informative marginal posterior densities for population size for both demes as shown in Figure 5.

**Figure 6:**
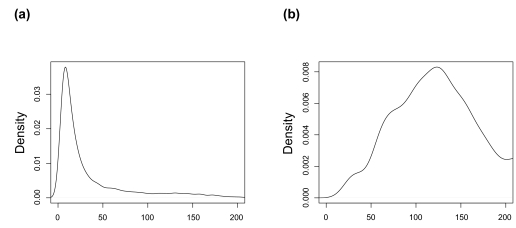
Marginal posterior density for *λ̂* from two runs, when there are a few sequences collected from the ghost deme for high migration. On the left there is a well defined mode and generally over estimates the true migration rate (Truth *λ* = 5) On the right low migration rates are excluded but otherwise it is uninformative and this is a general result for higher migration rates (Truth *λ* = 50).

There is a transition between low and high migration rates with regard to estimation of migration rates however. At *λ* = 20 the marginal migration posteriors often look like Figure 6(b) and we see they simply support any migration rate that is large. In this regime we note that population size estimates for the ghost deme become less informative and a long tail becomes the common distribution. However, the mode is still well defined for *θ*_1_ and we are able to make informed estimates of population sizes.

At low migration rates *λ* = 5, migration is somewhat resolved with a clearmode and overestimates the true value as previously reported and shown in Figure 6(b). With these reasonably well defined migration posteriors, particularly at low migration rates, there is larger variance for the estimate of population size of the ghost deme due to a smaller number of sequences and lack of data at other sample times.

From visual inspection of the traces it was observed that there were significant mixing issues for some datasets with large migration (*λ* ≥ 20). The nature of the slow mixing was identical to that already discussed in Section 3.0.2. ESS for some runs were therefore quite small (200–500) and long run times were often required. This further illustrates the point that a better MCMC proposal kernel is needed to improve the mixing and allow larger datasets to be practical.

## Discussion

In this paper we have looked at the ability to infer population size and migration rate using a coalescent-based Bayesian MCMC approach when we have serial samples from just one deme, while the true underlying population structure includes a hidden or ghost deme with migration between them. The case when we have samples from both demes only at the last time point was also presented. We also consider the related problem of estimating population size assuming a ghost deme when the true model does not contain this unobserved deme. It was found that in the former case, where there is a hidden deme when it is not taken into account, it overestimates the population size. As migration rates tend to large values, the estimate of population size tends to the sum of the subpopulations as we expect. For the opposite case, where the hidden deme is incorrectly included, *θ̂*_1_ is strongly underestimated and confidence intervals were very large. We did identify that mixing with respect to zero migration was poor, but this alone could not explain all of the bias.

In all cases, estimating under the correct model gave informative inference and reduced biases as compared to estimation under the incorrect model. The inclusion of a ghost deme when there is such a ghost deme effectively removes the dependence of estimated population size with migration rate. However, there is still a slight downward bias in the mean estimators, and migration estimates were very diffuse. The true value for *θ*_1_ was within the 95% HPD interval as expected and we could reject *θ̂*_1_ = *θ*_1_ + *θ*_2_ 50% of the time. This compares to the base case of estimating with no ghost deme when one does not exist, where we reject the larger *θ̂*_1_ about 30% of the time (First column of Table 2 for case I).

Adding a small number of sequences at the last time point for the ghost deme made a substantial difference. An instance where this type of data may arise is with HIV populations within hosts. Often we can only sample some tissues at autopsy and we may have no sequence data from these tissues at earlier sample times. The bias that was observed was effectively removed for low migration rates and reduced for large migration rates. At low migration rates estimation can be acceptable, while at high migration all we may be able to do is rule out low migration rates.

We have established that in general for the structured coalescent, we need to be accurate with the population structure to avoid misleading results. But we can’t always be sure of what the “correct” model is. However, there is no available test that compares different island-population models. Surprisingly a direct Bayesian model averaging approach or even any coalescent based approach to this question has not been carried out anywhere in the field. We suggest Bayesian model averaging as a way of addressing this problem.

Bayesian Model Averaging (BMA) in a MCMC setting is implemented by adding a move that jumps between models with the appropriate acceptance probability (Hoeting et al. 1999). In effect, we treat the current model as part of the state space or as one of the estimated parameters. More complicated model averaging can allow moves between models with different numbers of parameters (Green, 1995). Generally these types of moves are more difficult to develop. One possible genealogy move for our case, is the move between panmictic and subdivided populations. To move from the subdivided population model to panmictic model, we simply remove the migration events and relable the leaf nodes to be in the same deme. However, the opposite move is more difficult. A number of legal migration events need to be generated over the genealogy and the leaves again relabelled. This move must be capable of producing every possible migration event pattern on the genealogy, and the probability must be calculable so the acceptance probability can be evaluated. This type of move is particularly generic in that moves between 2 to 3 demes and vice versa are also possible by first removing all the migration labels and just regenerating a legal migration history. The recolour tree move proposed in Ewing and Rodrigo (2006) does this and gives reasonable acceptance rates.

BMA provides more flexibility when compared to alternatives, for example, Bayes factors. When we wish to just have an estimate of the population size, we can marginalize the posterior distribution across models and get a combined estimate. The difference in bias between the ghost data estimated without the ghost deme, compared to estimation with a ghost deme when *λ* = 0 suggested that there are some distributional differences between the two cases. Thus BMA could possibly work well for this type of problem and is the next logical step for this method. We are currently working on a model averaging version of the code.

## Figures and Tables

**Table 1:** Summary of the results from this study and from Beerli (2004). We have excluded migration results from Beerli (2004). Models used for data generation and estimation are shown horizontally and vertically respectively. When *λ* = 0 there is no migration from or to the ghost deme and it is effectively excluded. Results show that the two studies agree qualitatively and illustrate a general trend that estimation with the incorrect demographic model leads to poor performance of the estimators.

Data Generation Model	Estimation Model	
	*λ* = 0 (No Ghost Deme)	*λ* > 0 (Ghost Deme)
*λ***=0**	Case I(a)	Case I(b)
	Beerli:NA	Beerli: *θ̂*_1_ underestimates the truth.
	This Paper: *θ̂*_1_ estimates accurately in this base case.	This Paper: *θ̂*_1_ has a much larger downward bias than in Beerli’s results.

*λ***>0**	Case II(a)	Case II(b)
	Beerli: *θ̂*_1_ overestimates, with the bias increasing with increasing migration rate.	Beerli: *θ̂*_1_ estimates reasonably accurately even with higher migrations.
	This Paper: *θ̂*_1_ is also overestimated and tends to *θ*_1_ + *θ*_2_ with increasing migration rate.	This Paper: *θ̂*_1_ estimation is again reasonably accurate even for high migration. However, mixing becomes difficult at very high migration rates and long run times are required.

**Table 2:** The results showing the means of the modes for the different cases and migration rates. A clear trend is observed when the hidden deme is not taken into account (case I), and we see the truth is rejected more than expected even for low migration rates. As migration tends to large values the estimate of *θ̂*_1_ tends to *θ*_1_ + *θ*_2_. However, the trend of increasing estimates of *θ̂*_1_ with increasing migration rate does not hold when we take the ghost deme into account (case II). Once we have some samples from the ghost deme at the last sampling time, the performance improves dramatically. Estimates for *θ̂*_2_ are included and provides reasonable estimates with a slightly larger standard deviation. It is noted that we accept the truth as expected.

Migration rate	0	1	2	5	10	20	50
case I							
${\bar{\hat{\theta }}}_{1}$	0.102	0.142	0.1621	0.169	0.181	0.217	0.202
95% HPD 0.1	100%	75%	67%	61%	49%	45%	34%
95% HPD 0.2	30%	62%	84%	86%	94%	94%	100%
case II							
${\bar{\hat{\theta }}}_{1}$	0.02	0.0997	0.0697	0.0702	0.0688	0.082	0.0705
σ̄*_θ_*__1__	-	0.0433	0.0272	0.029	0.025	0.0122	0.0297
95% HPD 0.1	100%	82%	88%	85%	92%	90%	96%
95% HPD 0.2	30%	31%	31%	36%	53%	58%	55%
case III							
${\bar{\hat{\theta }}}_{1}$	-	0.093	0.0981	0.0913	0.0878	0.0867	0.1
σ̄*_θ_*__2__	-	0.02	0.032	0.0268	0.03	0.0433	0.136
${\bar{\hat{\theta }}}_{2}$	-	0.105	0.105	0.102	0.0923	0.106	0.108
σ̄*_θ_*__2__	-	0.04	0.0468	0.0462	0.0562	0.095	0.135
95% HP 0.1*θ̂*_1_	-	96%	94%	94%	94%	86%	86%
95% HPD 0.1 *θ̂*_2_	-	98%	96%	96%	92%	92%	86%

## Footnotes

1 The code supports extending any MCMC chain from where it stops. It records the machine state in a file every twenty minutes and then at the end of a run. It is then easy to “restart” a run from where it stopped and make it longer.

## References

[b1-ebo-02-253] Beerli P (2004). Effect of unsampled populations on the estimation of population sizes and migration rates between sampled populations. Molecular Ecology.

[b2-ebo-02-253] Beerli P, Felsenstein J (1999). Maximum-likelihood estimation of migration rates and effective population numbers in two populations using a coalescent approach. Genetics.

[b3-ebo-02-253] Beerli P, Felsenstein J (2001). Maximum likelihood estimation of a migration matrix and effective population sizes in n subpopulations by using a coalescent approach.. Proc. Natl. Soc. Sci.

[b4-ebo-02-253] Drummond AJ, Nicholls GK, Rodrigo AG, Solomon W (2002). Estimating mutation parameters, population history and genealogy simultaneously from temporally spaced sequence data. Genetics.

[b5-ebo-02-253] Ewing GB, Nicholls GK, Rodrigo AG (2004). Using temporally spaced sequences to simultaneously estimate migration rates, mutationrate and population sizes in measurably evolving populations. Genetics.

[b6-ebo-02-253] Ewing GB, Rodrigo AG (2006). Coalescent-based estimation of population parameters when the number of demes changes over time.. Mol. Biol. Evol.

[b7-ebo-02-253] Felsenstein J (1992). Estimating effective population size from samples of sequences: a bootstrap Monte Carlo intergration method.. Genet. Res.

[b8-ebo-02-253] Geyer CJ (1992). Practical Markov Chain Monte Carlo.. Statist. Sci.

[b9-ebo-02-253] Green PJ (1995). Reversible jump Markov Chain Monte Carlo computation and Bayesian model determination. Biometrika.

[b10-ebo-02-253] Hoeting JA, Madigan D, Raftery AE, Volinsky CT (1999). Bayesian model averaging: a tutorial.. Statist. Sci.

[b11-ebo-02-253] Hudson RR (1990). Gene genealogies and the coalescent process.. Oxford Surv. Evol. Biol.

[b12-ebo-02-253] Kingman J (1982a). The coalescent. Stoch.. Proc. Appl.

[b13-ebo-02-253] Kingman J (1982b). On the genealogy of large populations.. J. Appl. Probab.

[b14-ebo-02-253] Nagylaki T (1980). The strong-migration limit in geographically structured populations.. J. Math. Biol.

[b15-ebo-02-253] Nickle DC, Jensen MA (2003). Evolutionary indicators of human immunodeficiency virus type 1 reservoirs and compartments.. J. Virol.

[b16-ebo-02-253] Notohara M (1993). The strong-migration limit for the genealogical process in geographically structured populations.. J. Math. Biol.

[b17-ebo-02-253] Wong JK, Cignacio C (1997). In vivo compartmentalization of human immunodeficiency virus: evidence from the examination of pol sequences from autopsy tissues.. J. Virol.

